# Application of Optimal Control to Influenza Pneumonia Coinfection with Antiviral Resistance

**DOI:** 10.1155/2020/5984095

**Published:** 2020-03-10

**Authors:** Caroline W. Kanyiri, Livingstone Luboobi, Mark Kimathi

**Affiliations:** ^1^Department of Mathematics, Pan African University Institute of Basic Sciences, Technology and Innovation, P.O. Box 62000-00200, Nairobi, Kenya; ^2^Institute of Mathematical Sciences, Strathmore University, P.O. Box 59857-00200, Nairobi, Kenya; ^3^Department of Mathematics, Machakos University, P.O. Box 139-90100, Machakos, Kenya

## Abstract

Influenza and pneumonia independently lead to high morbidity and mortality annually among the human population globally; however, a glaring fact is that influenza pneumonia coinfection is more vicious and it is a threat to public health. Emergence of antiviral resistance is a major impediment in the control of the coinfection. In this paper, a deterministic mathematical model illustrating the transmission dynamics of influenza pneumonia coinfection is formulated having incorporated antiviral resistance. Optimal control theory is then applied to investigate optimal strategies for controlling the coinfection using prevalence reduction and treatment as the system control variables. Pontryagin's maximum principle is used to characterize the optimal control. The derived optimality system is solved numerically using the Runge–Kutta-based forward-backward sweep method. Simulation results reveal that implementation of prevention measures is sufficient to eradicate influenza pneumonia coinfection from a given population. The prevention measures could be social distancing, vaccination, curbing mutation and reassortment, and curbing interspecies movement of the influenza virus.

## 1. Introduction

Clinical evidence points out that infection with a particular combination of pathogens results in an aggravated infection with more severe clinical outcome compared with infection with either pathogen alone [1]. This is specially true for influenza virus and bacterium *Streptococcus pneumoniae* [2–4]. Influenza and pneumonia each contributes greatly to the global burden of morbidity and leads to high death toll, typically over a relatively short period of time [5–8]. *Streptococcus pneumoniae*, *Haemophilus influenzae*, and *Staphylococcus aureus* are the most common causes of pneumonia, the chief being *Streptococcus pneumoniae* [9, 10]. Coinfection resulting from influenza virus and *Streptococcus pneumoniae* further increases morbidity and mortality and is a major public health concern. These two pathogens rank among the chief pathogens affecting humans, and their ability to work together presents a major threat to world health [11]. Coinfection greatly impairs the host's immune system, increases antibacterial therapy intolerance, and can be detrimental to the diagnosis of the disease [12]. According to [13], it can be difficult to identify influenza patients experiencing bacterial coinfections due to symptom overlap of influenza and bacterial infections. In [14], it is indicated that a strong index of suspicion and additional diagnostic testing may be required for diagnosis and treatment of the infections.

The morbidity, mortality, and economic burden resulting from the lethal synergism that exists between influenza virus and pneumococcus are of major concern globally. The catastrophic 1918 influenza pandemic is an extreme example of the impact that results from this cooperative interaction [11]. Lung tissue samples examined from those who died during this pandemic revealed that the majority of deaths were as a result of secondary bacterial pneumonia. Data from the subsequent 1957, 1968, and 2009 influenza pandemics are consistent with these findings [15, 16]. In addition, during seasonal influenza outbreaks, coinfections resulting from influenza and *Streptococcus pneumoniae* have been associated with high morbidity and mortality rates [17–19]. According to [11], influenza virus alters the lungs in such a way that predisposes them to invasion by pneumococcus rendering a mild influenza infection severe or even fatal. This could be through several ways such as epithelial damage, changes in airway function, upregulation and exposure of receptors, dampening of the immune response, or amplification of inflammation. Several studies have been carried out to investigate the time course of susceptibility to *Streptococcus pneumoniae* after influenza virus infection. Results revealed that on average, individuals developed coinfection within 6 days after influenza virus infection [20–23].

Emergence of drug resistance, which has become a global concern, complicates influenza pneumonia coinfection even more. Drug resistance refers to the ability of disease-causing agents to resist the effects of drugs, thereby making the conventional treatment procedure ineffective. This leads to persistence of infections in the body, hence increasing the risk of spread to other individuals [24, 25]. The evolution of drug resistance is accelerated by overuse and misuse of antimicrobials, inappropriate use of antimicrobials, subtherapeutic dosing, and patient noncompliance with the recommended course of treatment [26]. There are two classes of antiviral drugs that are approved to treat influenza infections; these are M2 ion-channel inhibitors and neuraminidase (NA) inhibitors. However, due to antiviral drug resistance in influenza virus, neuraminidase (NA) inhibitors are the only class of antiinfluenza drugs currently in use as most of the circulating influenza viruses have acquired resistance to M2 ion-channel inhibitors [27, 28]. Moreover, many circulating influenza viruses have also acquired resistance to neuraminidase (NA) inhibitors [28, 29] raising an alarm in the health sector. Drug resistance continues to threaten effective prevention and treatment of influenza pneumonia coinfection. In addition, the cost of health care for patients with resistant infections is much higher than care for patients with nonresistant infections especially due to longer duration of illness.

Strategies such as vaccination, isolation, and treatment among others are necessary in order to curb the spread of various infectious diseases. However, if they are not administered at the right time and in the right amount, curtailing the spread of the infectious diseases remains a difficult task. The application of optimal control is therefore very vital since it is a necessary tool in making decisions of the viable control strategies to be employed in eradicating diseases. Optimal control theory has been applied in the study of influenza, for instance, in [30–34], and in the study of pneumonia, for instance, in [35]. Given that influenza pneumonia coinfection is more disastrous than either of the single infections alone, this paper seeks to investigate optimal control strategies for the influenza pneumonia coinfection and in the emergence of antiviral resistance.

## 2. Model Formulation

**Figure 1:**
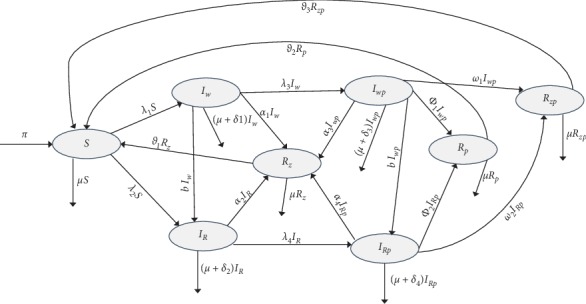
Schematic diagram showing population flow between different epidemiological classes for influenza pneumonia coinfection.

The model presented in this paper has the total population subdivided into eight compartments. These are susceptible (S), infected with wild-type influenza strain (*I*_*w*_), infected with resistant influenza strain (*I*_*R*_), infected with both wild-type influenza strain and pneumonia (*I*_*wp*_), infected with both resistant influenza strain and pneumonia (*I*_*Rp*_), recovered from influenza (*R*_*z*_), recovered from pneumonia (*R*_*p*_), and recovered from both influenza and pneumonia (*R*_*zp*_). In the model, individuals are first infected with influenza virus and then contract bacterial pneumonia. An assumption is made that there is no primary bacterial pneumonia infection. Individuals enter the population via immigration at the rate of *π*, and all recruited individuals are assumed to be susceptible. The susceptible can either become infected with wild-type influenza strain or resistant influenza strain at the rates of *λ*_1_ and *λ*_2_, respectively, where *λ*_1_=*β*_*w*_(*I*_*w*_+*η*_1_*I*_*wp*_), while $\lambda_{2}=\bar{\beta_{r}}\left(I_{R}+\eta_{2}I_{Rp}\right)$, where $\bar{\beta_{r}}=f\left(\beta_{r},b\right)$, in which parameter *b* refers to the rate of developing drug resistance while parameters *β*_*w*_ and *β*_*r*_ refer to the transmission rate of wild-type strain and resistant strain, respectively. Parameters *η*_1_ and *η*_2_ are modification parameters accounting for the relative infectiousness of individuals in *I*_*wp*_ and *I*_*Rp*_ classes in comparison with those in *I*_*w*_ and *I*_*R*_, respectively. Those infected with influenza wild-type strain can recover at a rate of *α*_1_ while those infected with influenza resistant strain recover at the rate of *α*_2_. The model incorporates development of antiviral resistance; hence, individuals infected with influenza wild-type strain progress to the *I*_*R*_ class at a rate of *b*. Those infected with influenza wild-type strain and those infected with influenza resistant strain can contract secondary bacterial pneumonia at the rate of *λ*_3_ with the force of infection of pneumonia being *β*_*p*_(*I*_*wp*_+*I*_*Rp*_). Parameter *β*_*p*_ refers to the transmission rate of pneumonia. Individuals in *I*_*w*_ and *I*_*R*_ classes also suffer disease-induced death at the rates of *δ*_1_ and *δ*_2_, respectively. Individuals infected with both the wild-type influenza strain and pneumonia and those infected with both resistant strain and pneumonia can recover from either influenza alone at rates of *α*_3_ and *α*_4_, respectively, or pneumonia alone at rates of *ϕ*_1_ and *ϕ*_2_, respectively, or recover from both influenza and pneumonia at rates of *ω*_1_ and *ω*_2_, respectively. This means that the *R*_*z*_ class contains some individuals from *I*_*wp*_ and *I*_*Rp*_ classes that have only recovered from influenza but may still have pneumonia. *R*_*z*_ class is however considered noninfectious given that bacterial pneumonia is considered weakly infectious after antimicrobials are administered. On the other hand, the *R*_*p*_ class contains some individuals from *I*_*wp*_ and *I*_*Rp*_ classes that have only recovered from pneumonia but may still have influenza. Given that the infectious period of influenza is about seven days after symptoms onset, *R*_*p*_ is considered noninfectious. Individuals in *I*_*wp*_ and *I*_*Rp*_ suffer disease-induced death at the rate of *δ*_3_ and *δ*_4_, respectively. Those infected with both wild-type influenza strain and pneumonia could also develop antiviral resistance and progress to *I*_*Rp*_ class at a rate of *b*. Individuals who have recovered from influenza, pneumonia, and both influenza and pneumonia lose their immunity and become susceptible again at rates of *ϑ*_1_, *ϑ*_2_, and *ϑ*_3_, respectively. Individuals in all the epidemiological compartments suffer natural death at the rate of *μ*.  Figure 1 shows the population flow between the different compartments.

### 2.1. Model Equations

Given the dynamics described in Figure 1, the following system of nonlinear ordinary differential equations, with nonnegative initial conditions, describes the dynamics of influenza pneumonia coinfection:

(1) $$\begin{array}{c} \left.\begin{array}{c} \frac{\text{d}S}{\text{d}t}=\pi +\vartheta_{1}R_{z}\left(t\right)+\vartheta_{2}R_{p}\left(t\right)+\vartheta_{3}R_{zp}\left(t\right)-\left(\lambda_{1}+\lambda_{2}+\mu \right)S\left(t\right)\\ \frac{\text{d}I_{w}}{\text{d}t}=\lambda_{1}S\left(t\right)-\left(\lambda_{3}+b+\alpha_{1}+\mu +\delta_{1}\right)I_{w}\left(t\right)\\ \frac{\text{d}I_{R}}{\text{d}t}=\lambda_{2}S\left(t\right)+bI_{w}\left(t\right)-\left(\lambda_{3}+\alpha_{2}+\mu +\delta_{2}\right)I_{R}\left(t\right)\\ \frac{\text{d}I_{wp}}{\text{d}t}=\lambda_{3}I_{w}\left(t\right)-\left(\alpha_{3}+\phi_{1}+\omega_{1}+b+\mu +\delta_{3}\right)I_{wp}\left(t\right)\\ \frac{\text{d}I_{Rp}}{\text{d}t}=\lambda_{3}I_{R}\left(t\right)+bI_{wp}\left(t\right)-\left(\alpha_{4}+\phi_{2}+\omega_{2}+\mu +\delta_{4}\right)I_{Rp}\left(t\right)\\ \frac{\text{d}R_{z}}{\text{d}t}=\alpha_{1}I_{w}\left(t\right)+\alpha_{2}I_{R}\left(t\right)+\alpha_{3}I_{wp}\left(t\right)+\alpha_{4}I_{Rp}\left(t\right)-\left(\vartheta_{1}+\mu \right)R_{z}\left(t\right)\\ \frac{\text{d}R_{p}}{\text{d}t}=\phi_{1}I_{wp}\left(t\right)+\phi_{2}I_{Rp}\left(t\right)-\left(\vartheta_{2}+\mu \right)R_{p}\left(t\right)\\ \frac{\text{d}R_{zp}}{\text{d}t}=\omega_{1}I_{wp}\left(t\right)+\omega_{2}I_{Rp}\left(t\right)-\left(\vartheta_{3}+\mu \right)R_{zp}\left(t\right) \end{array}\right\}, \end{array}$$

where

(2) $$\begin{array}{c} \lambda_{1}=\beta_{w}\left(I_{w}+\eta_{1}I_{wp}\right),\\ \lambda_{2}=\bar{\beta_{r}}\left(I_{R}+\eta_{2}I_{Rp}\right),\\ \lambda_{3}=\beta_{p}\left(I_{wp}+I_{Rp}\right). \end{array}$$

We assume that all the model parameters are positive and the initial conditions of model system (1) are given by

(3) $$\begin{array}{c} S\left(0\right)>0,\\ I_{w}\left(0\right)\geq 0,\\ I_{R}\left(0\right)\geq 0,\\ I_{wp}\left(0\right)\geq 0,\\ I_{Rp}\left(0\right)\geq 0,\\ R_{z}\left(0\right)\geq 0,\\ R_{p}\left(0\right)\geq 0,\\ R_{zp}\left(0\right)\geq 0. \end{array}$$

Table 1 gives the description of the various parameters used in the model.

## 3. Coinfection Model with Controls

In order to identify optimal control strategies that minimize the number of infected individuals and the cost of implementing the controls, a mathematical optimal control problem is formulated and analysed. Influenza pneumonia coinfection model (1) is extended to include time-dependent control measures. Let *u*_*i*_(*t*), *i*=1,2,3,4,5, represent the time-dependent controls whereby controls *u*_1_(*t*), *u*_2_(*t*), and *u*_3_(*t*) relate to prevalence reduction of wild-type influenza strain, resistant influenza strain, and pneumonia, respectively. The infection prevalence reduction could be through social distancing, vaccination, curbing mutation and reassortment, and curbing interspecies movement of the influenza virus. Controls *u*_4_(*t*) and *u*_5_(*t*) relate to treatment of the wild-type and resistant influenza strains, respectively.

Time is specified and is given by *t* ∈ [0, *T*], where *T* is the final time. Given that there is a limitation on the maximum rate of treatment and prevalence reduction controls, bounded Lebesgue measurable control set is introduced and defined as

(4) $$\begin{array}{c} U=\left\{\left(u_{1},u_{2},u_{3},u_{4},u_{5}\right),0\leq u_{i}\leq u_{i}\max,i=1,2,3,4,5\right\}. \end{array}$$

To identify the required level of effort to control the infection, an objective functional to be minimized is given by

(5) $$\begin{array}{c} J\left(u_{1},u_{2},u_{3},u_{4},u_{5}\right)=\int_{0}^{T}\left(A_{1}I_{w}+A_{2}I_{R}+A_{3}I_{wp}+A_{4}I_{Rp}+\frac{1}{2}\sum_{i=1}^{5}q_{i}u_{i}^{2}\right)\text{d}t, \end{array}$$

subject to the differential system:

(6) $$\begin{array}{c} \left.\begin{array}{c} \frac{\text{d}S}{\text{d}t}=\pi +\vartheta_{1}R_{z}\left(t\right)+\vartheta_{2}R_{p}\left(t\right)+\vartheta_{3}R_{zp}\left(t\right)-\left(\left(1-u_{1}\right)\lambda_{1}+\left(1-u_{2}\right)\lambda_{2}+\mu \right)S\left(t\right)\\ \frac{\text{d}I_{w}}{\text{d}t}=\left(1-u_{1}\right)\lambda_{1}S\left(t\right)-\left(1-u_{3}\right)\lambda_{3}I_{w}\left(t\right)-\left(\alpha_{1}+u_{4}\right)I_{w}\left(t\right)-\left(b+\mu +\delta_{1}\right)I_{w}\left(t\right)\\ \frac{\text{d}I_{R}}{\text{d}t}=\left(1-u_{2}\right)\lambda_{2}S\left(t\right)+bI_{w}\left(t\right)-\left(1-u_{3}\right)\lambda_{3}I_{R}\left(t\right)-\left(\alpha_{2}+u_{5}\right)I_{R}\left(t\right)-\left(\mu +\delta_{2}\right)I_{R}\left(t\right)\\ \frac{\text{d}I_{wp}}{\text{d}t}=\left(1-u_{3}\right)\lambda_{3}I_{w}\left(t\right)-\left(\alpha_{3}+\phi_{1}+\omega_{1}+b+\mu +\delta_{3}\right)I_{wp}\left(t\right)\\ \frac{\text{d}I_{Rp}}{\text{d}t}=\left(1-u_{3}\right)\lambda_{3}I_{R}\left(t\right)+bI_{wp}\left(t\right)-\left(\alpha_{4}+\phi_{2}+\omega_{2}+\mu +\delta_{4}\right)I_{Rp}\left(t\right)\\ \frac{\text{d}R_{z}}{\text{d}t}=\left(\alpha_{1}+u_{4}\right)I_{w}\left(t\right)+\left(\alpha_{2}+u_{5}\right)I_{R}\left(t\right)+\alpha_{3}I_{wp}\left(t\right)+\alpha_{4}I_{Rp}\left(t\right)-\left(\vartheta_{1}+\mu \right)R_{z}\left(t\right)\\ \frac{\text{d}R_{p}}{\text{d}t}=\phi_{1}I_{wp}\left(t\right)+\phi_{2}I_{Rp}\left(t\right)-\left(\vartheta_{2}+\mu \right)R_{p}\left(t\right)\\ \frac{\text{d}R_{zp}}{\text{d}t}=\omega_{1}I_{wp}\left(t\right)+\omega_{2}I_{Rp}\left(t\right)-\left(\vartheta_{3}+\mu \right)R_{zp}\left(t\right) \end{array}\right\}. \end{array}$$

The coefficients *A*_1_, *A*_2_, *A*_3_, and *A*_4_ represent the costs associated with minimizing the infected population. On the other hand, the expression 1/2*q*_*i*_*u*_*i*_^2^ represents costs associated with controls *u*_*i*_, *i*=1,2,3,4,5. Quadratic expressions of the cost of controls are considered because costs follow a nonlinear representation especially at high intervention levels. The objective of minimizing the infected population and the cost of controls can be achieved through proper implementation of the controls over a time interval given by [0, *T*]. Therefore, we seek an optimal control set (*u*_1_^*∗*^, *u*_2_^*∗*^, *u*_3_^*∗*^, *u*_4_^*∗*^, *u*_5_^*∗*^) such that

(7) $$\begin{array}{c} J\left(u_{1}^{*},u_{2}^{*},u_{3}^{*},u_{4}^{*},u_{5}^{*}\right)=\underset{u_{1},u_{2},u_{3},u_{4},u_{5}}{\min}\left\{J\left(u_{1},u_{2},u_{3},u_{4},u_{5}\right)\right\}. \end{array}$$

The necessary conditions for the existence of an optimal solution come from Pontryagin's Maximum Principle [36]. This principle converts (5)–(6) into a problem of minimizing pointwise Hamiltonian *H*, with respect to (*u*_1_, *u*_2_, *u*_3_, *u*_4_, *u*_5_), and is obtained as

(8) $$\begin{array}{c} \left.\begin{array}{c} H=A_{1}I_{w}+A_{2}I_{R}+A_{3}I_{wp}+A_{4}I_{Rp}+\frac{1}{2}q_{1}u_{1}^{2}+\frac{1}{2}q_{2}u_{2}^{2}+\frac{1}{2}q_{3}u_{3}^{2}+\frac{1}{2}q_{4}u_{4}^{2}+\frac{1}{2}q_{5}u_{1}^{5}\\ +p_{1}\left(t\right)\left\{\pi +\vartheta_{1}R_{z}\left(t\right)+\vartheta_{2}R_{p}\left(t\right)+\vartheta_{3}R_{zp}\left(t\right)-\left(\left(1-u_{1}\right)\lambda_{1}+\left(1-u_{2}\right)\lambda_{2}+\mu \right)S\left(t\right)\right\}\\ +p_{2}\left(t\right)\left\{\left(1-u_{1}\right)\lambda_{1}S\left(t\right)-\left(1-u_{3}\right)\lambda_{3}I_{w}\left(t\right)-\left(\alpha_{1}+u_{4}\right)I_{w}\left(t\right)-\left(b+\mu +\delta_{1}\right)I_{w}\left(t\right)\right\}\\ +p_{3}\left(t\right)\left\{\left(1-u_{2}\right)\lambda_{2}S\left(t\right)+bI_{w}\left(t\right)-\left(1-u_{3}\right)\lambda_{3}I_{R}\left(t\right)-\left(\alpha_{2}+u_{5}\right)I_{R}\left(t\right)-\left(\mu +\delta_{2}\right)I_{R}\left(t\right)\right\}\\ +p_{4}\left(t\right)\left\{\left(1-u_{3}\right)\lambda_{3}I_{w}\left(t\right)-\left(\alpha_{3}+\phi_{1}+\omega_{1}+b+\mu +\delta_{3}\right)I_{wp}\left(t\right)\right\}\\ +p_{5}\left(t\right)\left\{\left(1-u_{3}\right)\lambda_{3}I_{R}\left(t\right)+bI_{wp}\left(t\right)-\left(\alpha_{4}+\phi_{2}+\omega_{2}+\mu +\delta_{4}\right)I_{Rp}\left(t\right)\right\}\\ +p_{6}\left(t\right)\left\{\left(\alpha_{1}+u_{4}\right)I_{w}\left(t\right)+\left(\alpha_{2}+u_{5}\right)I_{R}\left(t\right)+\alpha_{3}I_{wp}\left(t\right)+\alpha_{4}I_{Rp}\left(t\right)-\left(\vartheta_{1}+\mu \right)R_{z}\left(t\right)\right\}\\ +p_{7}\left(t\right)\left\{\phi_{1}I_{wp}\left(t\right)+\phi_{2}I_{Rp}\left(t\right)-\left(\vartheta_{2}+\mu \right)R_{p}\left(t\right)\right\}\\ +p_{8}\left(t\right)\left\{\omega_{1}I_{wp}\left(t\right)+\omega_{2}I_{Rp}\left(t\right)-\left(\vartheta_{3}+\mu \right)R_{zp}\left(t\right)\right\} \end{array}\right\}, \end{array}$$

where *p*_*i*_(*t*), *i*=1,…, 8 are the corresponding adjoint or costate variables to be determined by applying Pontryagin's Maximum Principle as in Theorem 1.

**Theorem 1 .**

Given optimal control set (*u*_1_^*∗*^, *u*_2_^*∗*^, *u*_3_^*∗*^, *u*_4_^*∗*^, *u*_5_^*∗*^) and the corresponding solutions *S*^*∗∗*^, *I*_*w*_^*∗∗*^, *I*_*R*_^*∗∗*^, *I*_*wp*_^*∗∗*^, *I*_*Rp*_^*∗∗*^, *R*_*z*_^*∗∗*^, *R*_*p*_^*∗∗*^,  and *R*_*zp*_^*∗∗*^ of system (6) that minimizes *J*(*u*_1_, *u*_2_, *u*_3_, *u*_4_, *u*_5_) over *U*, there exist adjoint variables *p*_*i*_, *i*=1,…, 8, such that

(9) $$\begin{array}{c} \frac{\text{d}p_{1}}{\text{d}t}=-\frac{\partial H}{\partial S},\\ \frac{\text{d}p_{2}}{\text{d}t}=-\frac{\partial H}{\partial I_{w}},\\ \frac{\text{d}p_{3}}{\text{d}t}=-\frac{\partial H}{\partial I_{R}},\\ \frac{\text{d}p_{4}}{\text{d}t}=-\frac{\partial H}{\partial I_{wp}},\\ \frac{\text{d}p_{5}}{\text{d}t}=-\frac{\partial H}{\partial I_{Rp}},\\ \frac{\text{d}p_{6}}{\text{d}t}=-\frac{\partial H}{\partial R_{z}},\\ \frac{\text{d}p_{7}}{\text{d}t}=-\frac{\partial H}{\partial R_{p}},\\ \frac{\text{d}p_{8}}{\text{d}t}=-\frac{\partial H}{\partial R_{zp}}, \end{array}$$

with transversality condition *p*_*i*_(*T*)=0 for *i*=1,…, 8.

Evaluating (9) leads to the following adjoint system:

(10) $$\begin{array}{c} \left.\begin{array}{c} \frac{\text{d}p_{1}}{\text{d}t}=-p_{1}\left(-\mu -\left(1-u_{2}\right)\bar{\beta_{R}}\left(I_{R}+\eta_{2}I_{Rp}\right)-\left(1-u_{1}\right)\beta_{w}\left(I_{w}+\eta_{1}I_{wp}\right)\right)-\\ p_{3}\left(1-u_{2}\right)\bar{\beta_{R}}\left(I_{R}+\eta_{2}I_{Rp}\right)-p_{2}\left(1-u_{1}\right)\beta_{w}\left(I_{w}+\eta_{1}I_{wp}\right),\\ \frac{\text{d}p_{2}}{\text{d}t}=-A_{1}-p_{2}\left(-\alpha_{1}-b-\delta_{1}-\mu -\left(1-u_{3}\right)\beta_{p}\left(I_{wp}+I_{Rp}\right)+\left(1-u_{1}\right)\beta_{w}S-u_{4}\right)-bp_{3}\\ +p_{1}\left(1-u_{1}\right)\beta_{w}S-p_{6}\left(\alpha_{1}+u_{4}\right)-p_{4}\left(1-u_{3}\right)\beta_{p}\left(I_{wp}+I_{Rp}\right),\\ \frac{\text{d}p_{3}}{\text{d}t}=-A_{2}-p_{3}\left(-\alpha_{2}-\delta_{2}-\mu -\left(1-u_{3}\right)\beta_{p}\left(I_{wp}+I_{Rp}\right)+\left(1-u_{2}\right)\bar{\beta_{R}}S-u_{5}\right)+\\ p_{1}\left(1-u_{2}\right)\bar{\beta_{R}}S-p_{5}\left(1-u_{3}\right)\beta_{p}\left(I_{wp}+I_{Rp}\right)-p_{6}\left(\alpha_{2}+u_{5}\right),\\ \frac{\text{d}p_{4}}{\text{d}t}=-A_{3}-p_{5}\left(b+\left(1-u_{3}\right)\beta_{p}I_{R}\right)-p_{4}\left(-\alpha_{3}-b-\delta_{3}-\mu +\left(1-u_{3}\right)\beta_{p}I_{w}-\omega_{1}-\phi_{1}\right)\\ -\alpha_{3}p_{6}+p_{3}\left(1-u_{3}\right)\beta_{p}I_{R}+p_{1}\beta_{w}\eta_{1}S\left(1-u_{1}\right)-p_{2}\left(\beta_{w}\eta_{1}S\left(1-u_{1}\right)-\left(1-u_{3}\right)\beta_{p}I_{w}\right)-p_{8}\omega_{1}-p_{7}\phi_{1}\\ \frac{\text{d}p_{5}}{\text{d}t}=-A_{4}-\alpha_{4}p_{6}+\bar{\beta_{R}}\eta_{2}p_{1}S\left(1-u_{2}\right)-p_{3}\left(\bar{\beta_{R}}\eta_{2}S\left(1-u_{2}\right)-\left(1-u_{3}\right)\beta_{p}I_{R}\right)+p_{2}\left(1-u_{3}\right)\beta_{p}I_{w}\\ -p_{5}\left(-\alpha_{4}-\delta_{4}-\mu +\left(1-u_{3}\right)\beta_{p}I_{R}-\omega_{2}-\phi_{2}\right)-p_{4}\left(1-u_{3}\right)\beta_{p}I_{w}-p_{8}\omega_{2}-p_{7}\phi_{2}\\ \frac{\text{d}p_{6}}{\text{d}t}=-p_{6}\left(-\mu -\vartheta_{1}\right)-p_{1}\vartheta_{1}\\ \frac{\text{d}p_{7}}{\text{d}t}=-p_{7}\left(-\mu -\vartheta_{2}\right)-p_{1}\vartheta_{2}\\ \frac{\text{d}p_{8}}{\text{d}t}=-p_{8}\left(-\mu -\vartheta_{3}\right)-p_{1}\vartheta_{3} \end{array}\right\}. \end{array}$$

Following the results in [37, 38], the existence of optimal control is stated and proved using Theorem 2.

**Theorem 2 .**

There exists optimal controls (*u*_1_^*∗*^, *u*_2_^*∗*^, *u*_3_^*∗*^, *u*_4_^*∗*^, *u*_5_^*∗*^) which minimizes *J* over the region *U* satisfying the optimality condition:

(11) $$\begin{array}{c} u_{1}^{*}=\min\left\{\max\left\{0,\bar{u_{1}}\right\},u_{1}\max\right\},\\ u_{2}^{*}=\min\left\{\max\left\{0,\bar{u_{2}}\right\},u_{2}\max\right\},\\ u_{3}^{*}=\min\left\{\max\left\{0,\bar{u_{3}}\right\},u_{3}\max\right\},\\ u_{4}^{*}=\min\left\{\max\left\{0,\bar{u_{4}}\right\},u_{4}\max\right\},\\ u_{5}^{*}=\min\left\{\max\left\{0,\bar{u_{5}}\right\},u_{5}\max\right\}, \end{array}$$

where

(12) $$\begin{array}{c} {\bar{u}}_{1}=\frac{\left(\beta_{w}S^{**}\right)\left(p_{2}-p_{1}\right)\left(I_{w}^{**}+\eta_{1}I_{wp}^{**}\right)}{q_{1}},\\ {\bar{u}}_{2}=\frac{\left(\bar{\beta_{R}}S^{**}\right)\left(p_{3}-p_{1}\right)\left(I_{R}^{**}+\eta_{2}I_{Rp}^{**}\right)}{q_{2}},\\ {\bar{u}}_{3}=\frac{\beta_{p}\left(I_{wp}^{**}+I_{Rp}^{**}\right)I_{w}^{**}\left(p_{4}-p_{2}\right)+\left(p_{5}-p_{3}\right)\beta_{p}\left(I_{wp}^{**}+I_{Rp}^{**}\right)I_{R}^{**}}{q_{3}},\\ {\bar{u}}_{4}=\frac{\left(p_{2}-p_{6}\right)I_{w}^{**}}{q_{4}},\\ {\bar{u}}_{5}=\frac{\left(p_{3}-p_{6}\right)I_{R}^{**}}{q_{5}}. \end{array}$$

**Proof**

The Hamiltonian *H* is minimized with respect to the controls *u*_1_, *u*_2_, *u*_3_, *u*_4_, and *u*_5_ at the optimal control functions. This is done by differentiating the Hamiltonian function H with respect to each of the control variables on the set *U*; that is,

(13) $$\begin{array}{c} \frac{\partial H}{\partial u_{i}}=0. \end{array}$$

The following set of optimality conditions is thus obtained:

(14) $$\begin{array}{c} \frac{\partial H}{\partial u_{1}}=p_{1}S^{**}\beta_{w}\left(I_{w}^{**}+\eta_{1}I_{wp}^{**}\right)-p_{2}S^{**}\beta_{w}\left(I_{w}^{**}+\eta_{1}I_{wp}^{**}\right)+q_{1}u_{1}=0,\\ \frac{\partial H}{\partial u_{2}}=p_{1}S^{**}\bar{\beta_{R}}\left(I_{R}^{**}+\eta_{2}I_{Rp}^{**}\right)-p_{3}S^{**}\bar{\beta_{R}}\left(I_{R}^{**}+\eta_{2}I_{Rp}^{**}\right)+q_{2}u_{2}=0,\\ \frac{\partial H}{\partial u_{3}}=p_{3}\beta_{p}I_{R}^{**}\left(I_{wp}^{**}+I_{Rp}^{**}\right)-p_{5}\beta_{p}I_{R}^{**}\left(I_{wp}^{**}+I_{Rp}^{**}\right)+p_{2}\beta_{p}I_{w}^{**}\left(I_{wp}^{**}+I_{Rp}^{**}\right)\\ -p_{4}\beta_{p}I_{w}^{**}\left(I_{wp}^{**}+I_{Rp}^{**}\right)+q_{3}u_{3}=0,\\ \frac{\partial H}{\partial u_{4}}=-p_{2}I_{w}^{**}+p_{6}I_{w}^{**}+q_{4}u_{4}=0,\\ \frac{\partial H}{\partial u_{5}}=-p_{3}I_{R}^{**}+p_{6}I_{R}^{**}+q_{5}u_{5}=0, \end{array}$$

at *u*_1_ = *u*_1_^*∗*^, *u*_2_ = *u*_2_^*∗*^, *u*_3_ = *u*_3_^*∗*^, *u*_4_ = *u*_4_^*∗*^, and *u*_5_ = *u*_5_^*∗*^, respectively. Solving for *u*_1_^*∗*^, *u*_2_^*∗*^, *u*_3_^*∗*^, *u*_4_^*∗*^, and *u*_5_^*∗*^ and using the bounds for the controls in *U*, that is,

(15) $$\begin{array}{c} u_{i}^{*}=\left\{\begin{array}{cc} 0, & \text{if}\;\bar{u_{i}}\leq 0,\\ \bar{u_{i}}, & \text{if}\;0<\bar{u_{i}}<u_{i}\max\\ u_{i}\max, & \text{if}\;\bar{u_{i}}\geq u_{i}\max \end{array}\right. \end{array}$$

in compact notation yields,

(16) $$\begin{array}{c} u_{1}^{*}=\min\left\{\max\left(0,\frac{\beta_{w}S^{**}\left(p_{2}-p_{1}\right)\left(I_{w}^{**}+\eta_{1}I_{wp}^{**}\right)}{q_{1}}\right),u_{1}\max\right\},\\ u_{2}^{*}=\min\left\{\max\left(0,\frac{\bar{\beta_{R}}S^{**}\left(p_{3}-p_{1}\right)\left(I_{R}^{**}+\eta_{2}I_{Rp}^{**}\right)}{q_{2}}\right),u_{2}\max\right\},\\ u_{3}^{*}=\min\left\{\max\left(0,\frac{\beta_{p}\left(I_{wp}^{**}+I_{Rp}^{**}\right)I_{w}^{**}\left(p_{4}-p_{2}\right)+\left(p_{5}-p_{3}\right)\beta_{p}\left(I_{wp}^{**}+I_{Rp}^{**}\right)I_{R}^{**}}{q_{3}}\right),u_{3}\max\right\},\\ u_{4}^{*}=\min\left\{\max\left(0,\frac{\left(p_{2}-p_{6}\right)I_{w}^{**}}{q_{4}}\right),u_{4}\max\right\},\\ u_{5}^{*}=\min\left\{\max\left(0,\frac{\left(p_{3}-p_{6}\right)I_{R}^{**}}{q_{5}}\right),u_{5}\max\right\}. \end{array}$$

Next, the optimality system is obtained as

(17) $$\begin{array}{c} \frac{\text{d}S}{\text{d}t}=\pi +\vartheta_{1}R_{z}+\vartheta_{2}R_{p}+\vartheta_{3}R_{zp}-\left(\left(1-u_{1}^{*}\right)\lambda_{1}+\left(1-u_{2}^{*}\right)\lambda_{2}+\mu \right)S,\\ \frac{\text{d}I_{w}}{\text{d}t}=\left(1-u_{1}^{*}\right)\lambda_{1}S-\left(1-u_{3}^{*}\right)\lambda_{3}I_{w}-\left(\alpha_{1}+u_{4}^{*}\right)I_{w}-\left(b+\mu +\delta_{1}\right)I_{w},\\ \frac{\text{d}I_{R}}{\text{d}t}=\left(1-u_{2}^{*}\right)\lambda_{2}S+bI_{w}-\left(1-u_{3}^{*}\right)\lambda_{4}I_{R}-\left(\alpha_{2}+u_{5}^{*}\right)I_{R}-\left(\mu +\delta_{2}\right)I_{R},\\ \frac{\text{d}I_{wp}}{\text{d}t}=\left(1-u_{3}^{*}\right)\lambda_{3}I_{w}-\left(\alpha_{3}+\phi_{1}+\omega_{1}+b+\mu +\delta_{3}\right)I_{wp},\\ \frac{\text{d}I_{Rp}}{\text{d}t}=\left(1-u_{3}^{*}\right)\lambda_{4}I_{R}+bI_{wp}-\left(\alpha_{4}+\phi_{2}+\omega_{2}+\mu +\delta_{4}\right)I_{Rp},\\ \frac{\text{d}R_{z}}{\text{d}t}=\left(\alpha_{1}+u_{4}^{*}\right)I_{w}+\left(\alpha_{2}+u_{5}^{*}\right)I_{R}+\alpha_{3}I_{wp}+\alpha_{4}I_{Rp}-\left(\vartheta_{1}+\mu \right)R_{z},\\ \frac{\text{d}R_{p}}{\text{d}t}=\phi_{1}I_{wp}+\phi_{2}I_{Rp}-\left(\vartheta_{2}+\mu \right)R_{p},\\ \frac{\text{d}R_{zp}}{\text{d}t}=\omega_{1}I_{wp}+\omega_{2}I_{Rp}-\left(\vartheta_{3}+\mu \right)R_{zp},\\ \frac{\text{d}p_{1}}{\text{d}t}=-p_{1}\left(-\mu -\left(1-u_{2}\right)\bar{\beta_{R}}\left(I_{R}+\eta_{2}I_{Rp}\right)-\left(1-u_{1}\right)\beta_{w}\left(I_{w}+\eta_{1}I_{wp}\right)\right)\\ -p_{3}\left(1-u_{2}\right)\bar{\beta_{R}}\left(I_{R}+\eta_{2}I_{Rp}\right)-p_{2}\left(1-u_{1}\right)\beta_{w}\left(I_{w}+\eta_{1}I_{wp}\right),\\ \frac{\text{d}p_{2}}{\text{d}t}=-A_{1}-p_{2}\left(-\alpha_{1}-b-\delta_{1}-\mu -\left(1-u_{3}\right)\beta_{p}\left(I_{wp}+I_{Rp}\right)+\left(1-u_{1}\right)\beta_{w}S-u_{4}\right)-bp_{3}\\ +p_{1}\left(1-u_{1}\right)\beta_{w}S-p_{6}\left(\alpha_{1}+u_{4}\right)-p_{4}\left(1-u_{3}\right)\beta_{p}\left(I_{wp}+I_{Rp}\right),\\ \frac{\text{d}p_{3}}{\text{d}t}=-A_{2}-p_{3}\left(-\alpha_{2}-\delta_{2}-\mu -\left(1-u_{3}\right)\beta_{p}\left(I_{wp}+I_{Rp}\right)+\left(1-u_{2}\right)\bar{\beta_{R}}S-u_{5}\right)\\ +p_{1}\left(1-u_{2}\right)\bar{\beta_{R}}S-p_{5}\left(1-u_{3}\right)\beta_{p}\left(I_{wp}+I_{Rp}\right)-p_{6}\left(\alpha_{2}+u_{5}\right),\\ \frac{\text{d}p_{4}}{\text{d}t}=-A_{3}-p_{5}\left(b+\left(1-u_{3}\right)\beta_{p}I_{R}\right)-p_{4}\left(-\alpha_{3}-b-\delta_{3}-\mu +\left(1-u_{3}\right)\beta_{p}I_{w}-\omega_{1}-\phi_{1}\right)-\alpha_{3}p_{6}\\ +p_{3}\left(1-u_{3}\right)\beta_{p}I_{R}+p_{1}\beta_{w}\eta_{1}S\left(1-u_{1}\right)-p_{2}\left(\beta_{w}\eta_{1}S\left(1-u_{1}\right)-\left(1-u_{3}\right)\beta_{p}I_{w}\right)-p_{8}\omega_{1}-p_{7}\phi_{1},\\ \frac{\text{d}p_{5}}{\text{d}t}=-A_{4}-\alpha_{4}p_{6}+\bar{\beta_{R}}\eta_{2}p_{1}S\left(1-u_{2}\right)-p_{3}\left(\bar{\beta_{R}}\eta_{2}S\left(1-u_{2}\right)-\left(1-u_{3}\right)\beta_{p}I_{R}\right)+p_{2}\left(1-u_{3}\right)\beta_{p}I_{w}\\ -p_{5}\left(-\alpha_{4}-\delta_{4}-\mu +\left(1-u_{3}\right)\beta_{p}I_{R}-\omega_{2}-\phi_{2}\right)-p_{4}\left(1-u_{3}\right)\beta_{p}I_{w}-p_{8}\omega_{2}-p_{7}\phi_{2},\\ \frac{\text{d}p_{6}}{\text{d}t}=-p_{6}\left(-\mu -\vartheta_{1}\right)-p_{1}\vartheta_{1},\\ \frac{\text{d}p_{7}}{\text{d}t}=-p_{7}\left(-\mu -\vartheta_{2}\right)-p_{1}\vartheta_{2},\\ \frac{\text{d}p_{8}}{\text{d}t}=-p_{8}\left(-\mu -\vartheta_{3}\right)-p_{1}\vartheta_{3}. \end{array}$$

with *p*_*i*_(*T*) = 0 for *i* = 1,…, 8 and *S*(0) = *S*_0_, *I*_*w*_(0) = *I*_*w*0_, *I*_*R*_(0) = *I*_*R*0_, *I*_*wp*_(0) = *I*_*wp*0_, *I*_*Rp*_(0) = *I*_*Rp*0_, *R*_*z*_(0) = *R*_*z*0_, *R*_*p*_(0) = *R*_*p*0_,  and *R*_*zp*_(0) = *R*_*zp*0_.

## 4. Numerical Simulation

In this section, the optimal solution of optimality system (17) is investigated. Given that there are initial conditions for the state variables and terminal conditions for the adjoints, the optimality system is a two-point boundary value problem with separated boundary conditions at times *t*=0 and *t*=*T*. The forward-backward sweep method described in [39, 40] is hence used for the numerical solution of the optimal control problem. The method is named based on how the algorithm solves the problem's state and adjoint equations. The state variables are solved using the forward difference scheme while the adjoint variables are solved using the backward difference scheme. The solution iterative scheme makes an initial guess of the controls and using that guess solves the state system forward in time using a 4th-order Runge–Kutta scheme. Next, using the stored values of the controls and the solution of the state system, the adjoint system is solved backward in time using a 4th-order Runge–Kutta scheme. The RK4 method has to be adapted to account for solving backward in time. The controls are then updated using a convex combination of the previous controls and the values obtained using the characterizations. The updated controls are then used to repeat the solution of the state and adjoint systems. This process is repeated with values in the current iteration being tested for convergence against a user provided tolerance and depending on that, the algorithm either starts the process over again using the updated control or the algorithm terminates. The final approximations for the control, state, and adjoint systems are considered to be the solution to the optimal control problem.

### 4.1. Simulation Results

The numerical simulations were carried out using the MATLAB software and the parameter values in Table 2. There is however paucity of published estimates of morbidity and mortality rates resulting from influenza pneumonia coinfection in the middle- and low-income countries. On the other hand, in high-income, temperate countries where influenza surveillance has been done for years, these rates are well documented [47]; hence the data used in this research study are from these countries. The population under consideration is children <5 years and adults >60 years. Studies show that influenza pneumonia coinfection affects people of all ages; however, the morbidity and mortality rates of the high risk group which includes the immunocompromised, children <5 years, and adults >60 years are disproportionately high [9, 47, 48].

Most of the parameter values have a range as indicated in the references given in Table 2. Given that this research study focuses on a deterministic mathematical model to give an indication of the likely dynamics of influenza pneumonia coinfection, baseline parameter values were used for the simulations.

#### 4.1.1. Control by Treatment Only

**Figure 2:**
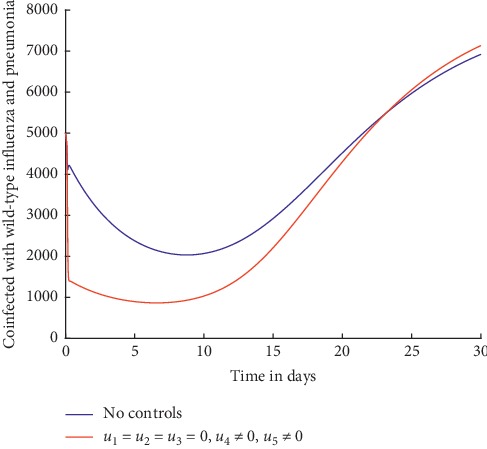
Individuals coinfected with wild-type influenza and pneumonia.

**Figure 3:**
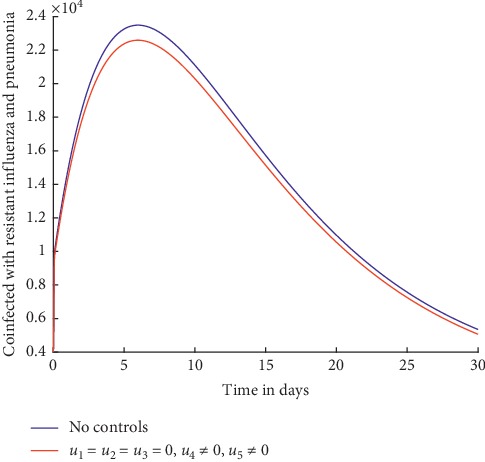
Individuals coinfected with resistant influenza and pneumonia.

Figures 2 and 3 show the effect of using treatment efforts only in an attempt to curb the spread of influenza pneumonia coinfection.

From Figure 2, it can be observed that with and without the treatment efforts, the wild-type influenza and pneumonia coinfection persists in the population.

Without any controls, it can be observed from Figure 3 that the number of individuals coinfected with resistant influenza and pneumonia increases initially and then decreases slowly but not to zero. With the treatment control strategies in place, the number of the infected individuals is lower but not significant and does not decrease to zero by day 30. This shows that use of treatment alone as a control strategy is not effective in curbing the spread of influenza pneumonia coinfection. As explained in details in [4, 49–51], treatment is often rendered ineffective because it is not always possible to administer the drugs at the right time and the diagnosis of the coinfection can be challenging because of timing of sample collection and false negative results when viruses replicate in the lower respiratory tract. In addition, treatment often aggravates development of resistance.

#### 4.1.2. Control by Prevention Measures Only

**Figure 4:**
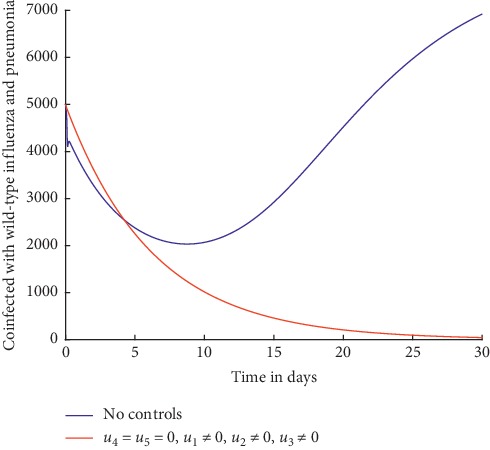
Individuals coinfected with wild-type influenza and pneumonia.

**Figure 5:**
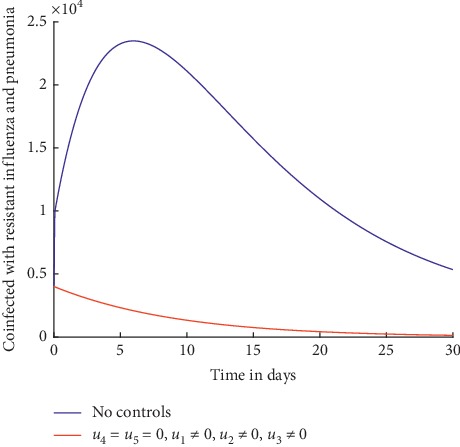
Individuals coinfected with resistant influenza and pneumonia.

Simulations are done when there is no control strategy in place and when there are controls involving prevention of wild-type influenza strain, prevention of influenza resistant strain, and prevention of pneumonia. Figures 4 and 5 show the results.

It can be observed from Figure 4 that when preventive efforts are implemented as control strategies, the number of individuals coinfected with wild-type influenza and pneumonia decreases to zero.

Similarly, from Figure 5, it can be observed that with preventive efforts as control strategies, the number of individuals coinfected with resistant influenza strain and pneumonia decreases drastically right from the beginning to zero by day 30.

The prevention measures help to reduce the transmission of the coinfection. Comparing Figures 2 and 3 with Figures 4 and 5, it can be observed that the preventive control strategies are more effective in curbing the spread of the coinfection compared to treatment control strategies.

#### 4.1.3. Control with Prevention and Treatment of Influenza

**Figure 6:**
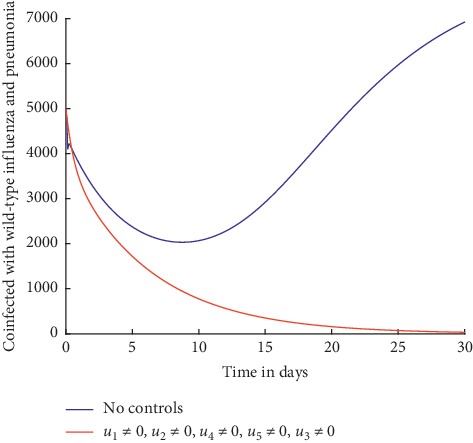
Individuals coinfected with wild-type influenza and pneumonia.

**Figure 7:**
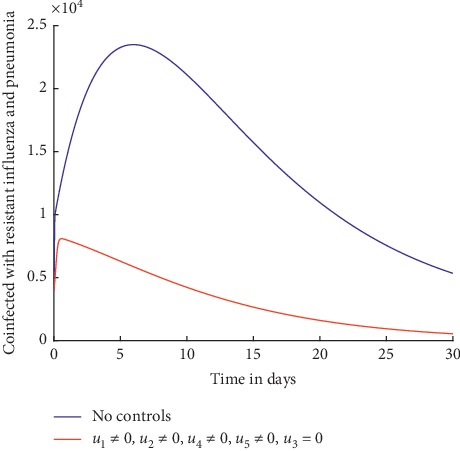
Individuals coinfected with resistant influenza and pneumonia.

Simulations are carried out to investigate the effect of implementing control strategies involving the prevention and treatment of influenza. Figures 6 and 7 show the results.

It can be observed from Figure 6 that with the prevention and treatment of influenza as control strategies, the number of individuals coinfected with wild-type influenza and pneumonia decreases right from the beginning and it is at zero by about day 25.

Figure 7 shows that with the implementation of these control strategies, the number of individuals coinfected with resistant influenza and pneumonia significantly decreases and it is inconsequential at day 30 implying that it is unlikely for the coinfection to persist in the population.

The prevention and treatment of influenza as control strategies aid in reducing the transmission and in treatment of those who are already infected; however, the treatment poses a danger of development of drug resistance. Therefore, caution should be taken during drug administration.

#### 4.1.4. Control with All Strategies

**Figure 8:**
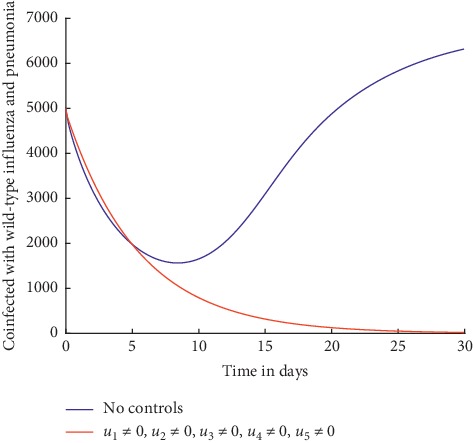
Individuals coinfected with wild-type influenza and pneumonia.

**Figure 9:**
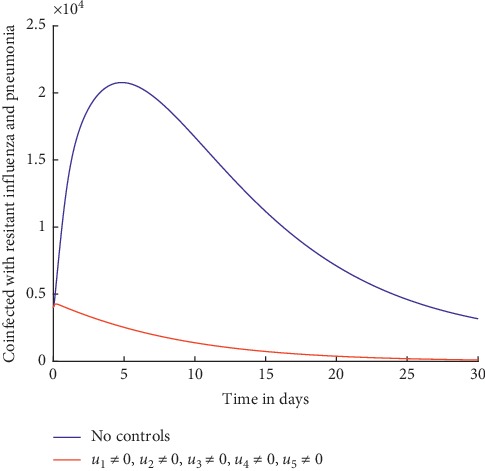
Individuals coinfected with resistant influenza and pneumonia.

When all the control strategies are applied, the number of infected individuals decreases as shown in Figures 8 and 9. From Figure 8, it can be observed that with all the control strategies in place, the number of individuals coinfected with wild-type influenza and pneumonia decreases to zero by day 30.

It can also be observed from Figure 9 that when all the control strategies are applied, the number of individuals coinfected with resistant influenza and pneumonia drastically decreases, and by about day 28, the number is already at zero.

## 5. Conclusion

As observed from Figures 2–9, different intervention mechanisms produce different results. Of great importance to public health is the control strategy that will help eradicate the diseases. Given that availability of resources is always a factor to consider when implementing control strategies, it is paramount to have a strategy with maximum benefit. From the results of this study, with the implementation of prevention measures only, which include and not limited to social distancing, vaccination, curbing mutation and reassortment, and curbing interspecies movement of the influenza virus, influenza pneumonia coinfection can be eradicated from a given population. Compared to the other control strategies investigated and discussed in Section 4.1, these preventive control strategies are more effective in curbing the spread of influenza pneumonia coinfection; hence, the public health sector and other stake holders could apply them to eradicate the coinfection within a given population.

## Figures and Tables

**Table 1:** Description of parameters used.

Parameters	Description
*π*	Recruitment rate
*β*_*w*_	Transmission rate of wild-type influenza strain
*β*_*r*_	Transmission rate of resistant influenza strain
*β*_*p*_	Transmission rate of pneumonia
*α*_1_	Recovery rate of influenza for individuals in *I*_*w*_ class
*α*_2_	Recovery rate of influenza for individuals in *I*_*R*_ class
*α*_3_	Recovery rate of influenza for individuals in *I*_*wp*_ class
*α*_4_	Recovery rate of influenza for individuals in *I*_*Rp*_ class
*ϕ*_1_	Recovery rate of pneumonia for individuals in *I*_*wp*_ class
*ϕ*_2_	Recovery rate of pneumonia for individuals in *I*_*Rp*_ class
*ω*_1_	Recovery rate of both influenza and pneumonia for individuals in *I*_*wp*_ class
*ω*_2_	Recovery rate of both influenza and pneumonia for individuals in *I*_*Rp*_ class
*ϑ*_1_, *ϑ*_2_, *ϑ*_3_	Rate of losing immunity for influenza, pneumonia, and influenza and pneumonia, respectively
*b*	Rate of developing antiviral resistance
*δ*_1_, *δ*_2_, *δ*_3_, *δ*_4_	Disease-induced death rates in *I*_*w*_,*I*_*R*_,*I*_*wp*_, and *I*_*Rp*_ classes, respectively
*μ*	Natural death rate

**Table 2:** Description and values of the different parameters used.

Parameter	Description	Value	Reference.
*π*	Recruitment rate	0.0381	Assumed
*β*_*w*_	Transmission rate of wild-type influenza strain	0.0102 day^−1^	Assumed
*β*_*r*_	Transmission rate of resistant influenza strain	0.00026 day^−1^	Assumed
*β*_*p*_	Transmission rate of pneumonia	0.000162 day^−1^	Reference [41]
*α*_1_	Recovery rate of influenza for individuals in *I*_*w*_ class	0.07143 day^−1^	Reference [42]
*α*_2_	Recovery rate of influenza for individuals in *I*_*R*_ class	0.0333 day^−1^	Assumed
*α*_3_	Recovery rate of influenza for individuals in *I*_*wp*_ class	0.04762 day^−1^	Reference [43].
*α*_4_	Recovery rate of influenza for individuals in *I*_*Rp*_ class	0.0222 day^−1^	Assumed
*ϕ*_1_	Recovery rate of pneumonia for individuals in *I*_*wp*_ class	0.033 day^−1^	Reference [41]
*ϕ*_2_	Recovery rate of pneumonia for individuals in *I*_*Rp*_ class	0.033 day^−1^	Reference [41]
*ω*_1_	Recovery rate of both influenza and pneumonia for individuals in *I*_*wp*_ class	0.0166 day^−1^	Assumed
*ω*_2_	Recovery rate of both influenza and pneumonia for individuals in *I*_*Rp*_ class	0.0166 day^−1^	Assumed
*ϑ*_1_	Rate of losing immunity for influenza	0.00833 day^−1^	Reference [44]
*ϑ*_2_	Rate of losing immunity for pneumonia	0.00833 day^−1^	Assumed
*ϑ*_3_	Rate of losing immunity for influenza pneumonia coinfection	0.00833 day^−1^	Assumed
*b*	Rate of developing antiviral resistance	0.0118	Assumed
*δ*_1_	Wild-type influenza strain-induced death rate	0.01	Reference [45]
*δ*_2_	Resistant influenza strain-induced death rate	0.021	Assumed
*δ*_3_	*I*_*wp*_ class disease-induced death rate	0.05	Assumed
*δ*_4_	*I*_*Rp*_ class disease-induced death rates	0.05	Assumed
$\frac{1}{\mu }$	Average human lifespan	70 × 365 days	Reference [46]

## Data Availability

The data used to support the findings of this study are included within the article.

## Supplementary Materials

Supplementary Materials The supplementary material is the source code (MATLAB) that was used to do the numerical simulations.
