# Supplementary material for: Application of Optimal Control to Influenza Pneumonia Coinfection with Antiviral Resistance
Source: Comput Math Methods Med. 2020 Mar 10;2020:5984095. doi: 10.1155/2020/5984095 (PMC7091548; doi:10.1155/2020/5984095)
Supplement: Supplementary Materials — The supplementary material is the source code (MATLAB) that was used to do the numerical simulations. [file 5984095.f1.pdf]

```
function optimControl()
clear all; clc;
K=100001;color='r';
t0=0;tf=30;h=(tf-t0)/(K-1);% h==dt stepsize
t=t0:h:tf;
S=zeros(K,1);Xw=zeros(K,1);Xr=zeros(K,1);Xwp=zeros(K,1);Xrp=zeros(K,1);Rz=zeros(K,1);Rp=zeros(K,1
);Rzp=zeros(K,1);
u1=zeros(K,1);u2=zeros(K,1);u3=zeros(K,1);u4=zeros(K,1);u5=zeros(K,1);
P1=zeros(K,1);P2=zeros(K,1);P3=zeros(K,1);P4=zeros(K,1);P5=zeros(K,1);P6=zeros(K,1);P7=zeros(K,1)
;P8=zeros(K,1);
S(1)=300000; Xw(1)=6000;Xr(1)=5000;Xwp(1)=5000;Xrp(1)=4000;Rz(1)=1500;Rp(1)=1000;Rzp(1)=800;
u1(1)=0;u2(1)=0;u3(1)=0;u4(1)=0;u5(1)=0;
P1(K-1:K)=0;P2(K-1:K)=0;P3(K-1:K)=0;P4(K-1:K)=0;P5(K-1:K)=0;P6(K-1:K)=0;P7(K-1:K)=0;P8(K-1:K)=0;
A1=1;A2=2;A3=3;A4=4;q1=10;q2=10;q3=10;q4=5;q5=5;beta1=0.0102;beta2=0.00026;b=0.0118;beta3=(1+b*b)
*beta2;H=0.0381;betap=0.000162;alpha1=0.07143;alpha2=0.0333;
alpha3=0.04762;alpha4=0.0222;phi1=0.0333;phi2=0.0333;omega1=0.0166;omega2=0.0166;theta1=0.0083;th
eta2=0.0083;theta3=0.0083;delta1=0.01;delta2=0.021;
delta3=0.05;delta4=0.05;miu=0.0000391;eta1=0.2;eta2=0.3;
for i=1:K-2
    S(i+1)=(S(i)+h*(H+theta1*Rz(i)+theta2*Rp(i)+theta3*Rzp(i)))/(1+h*((1-
u1(i))*beta1*(Xw(i)+eta1*Xwp(i))+(1-u2(i))*beta3*(Xr(i)+eta2*Xrp(i))+miu));
    Xw(i+1)=(Xw(i)+h*((1-u1(i))*beta1*eta1*Xwp(i)*S(i+1)))/(1+h*((1-
u3(i))*betap*(Xwp(i)+Xrp(i))+alpha1+u4(i)+b+miu+delta1-((1-u1(i))*beta1*S(i+1))));
    Xr(i+1)=(Xr(i)+h*((1-u2(i))*beta3*eta2*Xrp(i)*S(i+1)+b*Xw(i+1)))/(1+h*((1-
u3(i))*betap*(Xrp(i)+Xwp(i))+alpha2+u5(i)+miu+delta2-((1-u2(i))*beta3*S(i+1))));
    Xwp(i+1)=(Xwp(i)+h*((1-u3(i))*betap*Xw(i+1)*Xrp(i)))/(1+h*((alpha3+phi1+omega1+b+miu+delta3)-
((1-u3(i))*betap*Xw(i+1))));
    Xrp(i+1)=(Xrp(i)+h*(b*Xwp(i+1)+((1-
u3(i))*betap*Xwp(i+1)*Xr(i+1))))/(1+h*((alpha4+phi2+omega2+miu+delta4)-((1-
u3(i))*betap*Xr(i+1))));

Rz(i+1)=(Rz(i)+h*(((alpha1+u4(i))*Xw(i+1))+((alpha2+u5(i))*Xr(i+1))+alpha3*Xwp(i+1)+alpha4*Xrp(i+
1)))/(1+h*(theta1+miu));
    Rp(i+1)=(Rp(i)+h*(phi1*Xwp(i+1)+phi2*Xrp(i+1)))/(1+h*(theta2+miu));
    Rzp(i+1)=(Rzp(i)+h*(omega1*Xwp(i+1)+omega2*Xrp(i+1)))/(1+h*(theta3+miu));
    P1(K-i-1)=(P1(K-i)+h*((P3(K-i)*(1-u2(i))*beta3*(Xr(i+1)+eta2*Xrp(i+1)))+(P2(K-i)*(1-
u1(i))*beta1*(Xw(i+1)+eta1*Xwp(i+1)))))/(1+h*((1-u1(i))*beta1*(Xw(i+1)+eta1*Xwp(i+1))+(1-
u2(i))*beta3*(Xr(i+1)+eta2*Xrp(i+1))+miu));
    P2(K-i-1)=(P2(K-i)+h*(A1+b*P3(K-i)-P1(K-i-1)*(1-u1(i))*beta1*S(i+1)+P6(K-
i)*(alpha1+u4(i))+P4(K-i)*(1-u3(i))*betap*(Xwp(i+1)+Xrp(i+1))))/(1+h*((1-
u3(i))*betap*(Xwp(i+1)+Xrp(i+1))+alpha1+u4(i)+b+miu+delta1-((1-u1(i))*beta1*S(i+1))));
    P3(K-i-1)=(P3(K-i)+h*(A2-P1(K-i-1)*(1-u2(i))*beta3*S(i+1)+P6(K-i)*(alpha2+u5(i))+P5(K-i)*(1-
u3(i))*betap*(Xwp(i+1)+Xrp(i+1))))/(1+h*((1-
u3(i))*betap*(Xwp(i+1)+Xrp(i+1))+alpha2+u5(i)+miu+delta2-((1-u2(i))*beta3*S(i+1))));
    P4(K-i-1)=(P4(K-i)+h*(A3+P5(K-i)*(b+((1-u3(i))*betap*Xr(i+1)))+alpha3*P6(K-i)-P3(K-i-1)*((1-
u3(i))*betap*Xr(i+1))-P1(K-i-1)*(1-u1(i)*beta1*eta1*S(i+1))+P2(K-i-1)*((1-
u1(i))*beta1*eta1*S(i+1)-(1-u3(i))*betap*Xw(i+1))+P8(K-i)*omega1+P7(K-
i)*phi1))/(1+h*((alpha3+phi1+omega1+b+miu+delta3)-((1-u3(i))*betap*Xw(i+1))));
    P5(K-i-1)=(P5(K-i)+h*(A4+P4(K-i-1)*((1-u3(i))*betap*Xw(i+1))+alpha4*P6(K-i)-P2(K-i-1)*((1-
u3(i))*betap*Xw(i+1))-P1(K-i-1)*(1-u2(i))*beta3*eta2*S(i+1)-P3(K-i-1)*((1-
u2(i))*beta3*eta2*S(i+1)-(1-u3(i))*betap*Xrp(i+1))+P8(K-i)*omega2+P7(K-
i)*phi2))/(1+h*((alpha4+phi2+omega2+miu+delta4)-((1-u3(i))*betap*Xr(i+1))));
    P6(K-i-1)=(P6(K-i)+h*(P1(K-i-1)*theta1))/(1+h*(theta1+miu));
    P7(K-i-1)=(P7(K-i)+h*(P1(K-i-1)*theta2))/(1+h*(theta2+miu));
    P8(K-i-1)=(P8(K-i)+h*(P1(K-i-1)*theta3))/(1+h*(theta3+miu));
    M1(i+1)=((P2(K-i-1)-P1(K-i-1))*beta1*S(i+1)*(Xw(i+1)+eta1*Xwp(i+1)))/q1;
    M2(i+1)=((P3(K-i-1)-P1(K-i-1))*beta3*S(i+1)*(Xr(i+1)+eta2*Xrp(i+1)))/q2;
    M3(i+1)=(((P4(K-i-1)-P2(K-i-1))*betap*(Xwp(i+1)+Xrp(i+1))*Xw(i+1))/q3)+(((P5(K-i-1)-P3(K-i-
1))*betap*(Xwp(i+1)+Xrp(i+1))*Xr(i+1))/q3);
    M4(i+1)=((P2(K-i-1)-P6(K-i-1))*Xw(i+1))/q4;
    M5(i+1)=((P3(K-i-1)-P6(K-i-1))*Xr(i+1))/q5;
                                   u1(i+1)=min(1,max(0,M1(i+1)));
                                    u2(i+1)=min(1,max(0,M2(i+1)));
                                 u3(i+1)=min(1,max(0,M3(i+1)));
                                       u4(i+1)=min(1,max(0,M4(i+1)));
                                     u5(i+1)=min(1,max(0,M5(i+1)));
 end

Ss=S(:);Xws=Xw(:);Xrs=Xr(:);Xwps=Xwp(:);Xrps=Xrp(:);Rzs=Rz(:);Rps=Rp(:);Rzps=Rzp(:);u1s=u1(:);u2s
=u2(:);u3s=u3(:);u4s=u4(:);u5s=u5(:);
figure(1)
```

```
hold on
plot(t(1:K-2),Xrps(1:K-2),color)
hold on
xlabel('Time in days');
ylabel('Co-infected with resistant influenza and pneumonia');
end
```
